# Development and Implementation of a Global Health Elective with a Drug Discovery Game for Pharmacy Students

**DOI:** 10.3390/pharmacy5030049

**Published:** 2017-08-28

**Authors:** Jordan R. Covvey, Anthony J. Guarascio, Lauren A. O’Donnell, Kevin J. Tidgewell

**Affiliations:** Duquesne University School of Pharmacy, 600 Forbes Ave; Pittsburgh, PA 15282, USA; covveyj@duq.edu (J.R.C.); guarascioa@duq.edu (A.J.G.); odonnel6@duq.edu (L.A.O.)

**Keywords:** pharmacy education, global health, active learning, curriculum, elective course

## Abstract

Interest in global health education within the pharmacy curriculum has increased significantly in recent years. However, discussion of different models and methods to evaluate course structures are limited. The overall objective was to (1) describe the structure of our global health elective for pharmacy students, and (2) assess educational outcomes related to perceived/formal knowledge and attitudes associated with global health. Our elective was designed using a competency-centered approach to global health education, incorporating reflection, projects, service and game-learning. In addition to course assessments, a pre-post survey questionnaire assessing attitudes, knowledge perception, formalized knowledge and opinions was utilized. Overall, students demonstrated appropriate performance on course assessments, temporally improving throughout longitudinal projects. The survey demonstrated significant increases in knowledge perception as a result of the course; however, no change in formalized knowledge was evident through the survey assessment. Additionally, the incorporation of game-learning into the course was well received by students. Future iterations of the course will focus on utilization of different assessment methods to meet learning outcomes.

## 1. Introduction

### 1.1. Background

Global health is increasingly being featured within curricula in pharmacy education, primarily the result of growing interest and recognition of a wider role of pharmacists in the global environment [1,2]. Global health is widely defined as “an area for study, research, and practice that places a priority on improving health for all people worldwide … [it] emphasizes transnational health issues, determinants, and solutions; involves many disciplines within and beyond the health sciences and promotes interdisciplinary collaboration; and is a synthesis of population based prevention with individual-level clinical care” [3]. This definition is wide-reaching, including both clinical activities as well as science, focused abroad as well as in local communities, and serves both macro- and micro-levels of public health. Accordingly, introductory course offerings for health sciences students in this area are charged to provide broad curricular exposure, focused on development first as a ‘global citizen’, and secondarily, to build desired skill sets that contribute in a variety of settings. In this manuscript, we detail our creation of a global health elective for pharmacy students and our initial assessment of outcomes toward serving these goals.

### 1.2. Course Description

In our school, *Perspectives in Global Health* was created as an elective course offering in the third professional year of the PharmD curriculum, with the goal of providing a global view of pharmacy and understanding of healthcare, science and the role of the pharmacist within the world. The course is team-taught by four School of Pharmacy faculty members in different disciplines, including health outcomes research, clinical specialty in infectious diseases, basic science of infectious diseases, and basic science of drug discovery. The course follows a weekly seminar format with discussion led by a faculty member, supplemented with pre-readings. Students are engaged by in-class activities to promote thinking about the topics and increase student engagement. Major topic areas covered in the course were global health status and statistics, infectious and neglected tropical diseases, global health organizational infrastructure, culture/ethics, drug discovery, international science collaborations and immigration/poverty. Learning outcomes for students by the end of the course were to: (1) demonstrate a comprehensive understanding of global health issues pertinent to pharmacy and pharmaceutical science in the past, present and future, and (2) develop an enhanced awareness and ability to work with patients, healthcare and science within a widened world view. Learning outcomes were designed to emphasize student growth and recognition regarding their personal and professional roles, as well as awareness and willingness to engage in future activities to affect global health outcomes. To meet course objectives (Appendix A) and specific learning outcomes, students are engaged in several individual and group projects across the semester, which are described in the following sections and related back to course learning objectives.

#### 1.2.1. Neglected Tropical Diseases Eradication Plan

Students in teams are assigned an emerging and/or neglected disease and asked to submit a written proposal on the disease and how they could potentially eradicate it (learning objectives 1, 3, 4). In the class session prior to the assignment, examples of completed pathogen eradication programs (e.g., smallpox) and current pathogen eradication programs (e.g., poliovirus, Guinea worms) are discussed with emphasis on factors that made each disease amenable to eradication. Students must utilize basic science and clinical knowledge to present the causative organism, disease progression, points of intervention, risk factors, and barriers to treatment that might exist. Students are asked to develop a detailed, step-wise approach for an eradication plan, and to describe whether or not they believe eradication is feasible given current global resources available.

#### 1.2.2. Culture/Ethics Case Study Development

Students are individually provided two of eight patient-oriented case studies regarding ethical/cultural topics in global health (learning objectives 2 and 6). Students are asked to submit a written response that details the cultural/ethical issues at hand in the described scenarios, different approaches to approaching the scenarios, and final recommendations on how they would address the issue at hand.

#### 1.2.3. Country Health System Project

Students are asked to individually choose a country from a determined list and develop a short presentation detailing the country and a specific international health issue related to that country (learning objectives 1, 3, 5). Potential countries included Cuba, Ghana, Tanzania, India, Guyana, China, Brazil, Syria and Iran. The presentation was required to tie together the multiple topic areas covered by the course, including epidemiology of the health issue, healthcare structure of the country, culture/ethical issues to consider, potential drug utilization/development and role of organizational support for the issue. The project includes both an interim submission for faculty feedback, as well as final presentation to the class at the end of the semester.

#### 1.2.4. Volunteer Impact for a Local Global Health Organization

All students in the course are required to attend at least one volunteer session with a local organization, Global Links, a “medical relief and development organization dedicated to supporting health improvement initiatives in resource-poor communities and promoting environmental stewardship in the US healthcare system” [4] (learning objective 3). The volunteer session involves an introduction to the organization and the services they provide, and then an activity chosen by Global Links to meet whatever current needs they have, often including sorting and categorization of medical supply donations. This activity was developed to get students engaged with an opportunity in global health at the local level and to further understanding that actions in the local community can work to aid people around the world. This activity underpins longitudinal reinforcement of the idea throughout the course that global health does not necessarily require travel abroad, but rather, focuses on serving inequities in health across the globe.

#### 1.2.5. Patient Education Project for a Global Partner

Students additionally complete a group project partnered with another local organization focused on global health, namely the Espwa Foundation [5] (learning objectives 1, 3, 5, 6). This organization works in concert with other local partners in and around Cap-Haitien, Haiti with a goal to “identify needs and develop projects that empower and inspire hope”. Two of the faculty traveled with the organization to Haiti in 2016 to help identify potential ways to make a lasting impact on the community through medical relief and related activities. A Haitian physician partnering with the Espwa Foundation was opening a hospital for the underserved area and was in the process of arranging required resources for the facility. From this, a project was created in our course for students help create patient education materials on relevant medical topics that could be used in the new hospital setting for outreach. The assignment occurs longitudinally across the course as a multi-stage assignment to assist student progress. At the beginning of the semester, students choose small groups and are offered topic areas for their project focus; examples of topics in the past have included hygiene, maternal health, cholera, malaria, Zika, mental health, nutrition/malnutrition, and chronic disease. A collaborator with the Espwa Foundation provides a guest lecture discussing the organization and their work. Students are subsequently asked to research Haitian culture, healthcare system and available resources for their project for their first assignment. Next, they create a plan for information delivery and what they plan to communicate to patients on their topic. This is followed by a brief in-class presentation of their idea for feedback from the class and course instructors. The project concludes with creation and submission of their education project materials.

#### 1.2.6. Game of International Drug Discovery

Following a didactic session on drug discovery chemistry relevant to the global community, students are given two readings about international partnerships in drug discovery (learning objectives 7 and 8). To demonstrate how drug discovery is influenced by science, investment, biodiversity and country-specific priorities and development goals, students spend a class period playing the Game of International Drug Discovery, an investigator-designed learning activity. A full description of the game and rules is found in Appendix B. In short, students are placed into teams that represent fictitious countries with differing amounts of money, science capability and biodiversity at the start. Countries are asked to achieve a set of confidential goals through taking turns to invest in science, license drugs or explore biodiversity. Turns proceed through drawing of “drug” and “nature” cards which change the dynamics of each turn through situations such as outbreaks, natural disasters and scientific grants. Additionally, countries may interact through making agreements with each other. After the game, a short debriefing occurs in which the class discusses country-driven motivations and how the students felt about the game. Students finalize the activity by writing a short reflection on their self-assessed learning outcomes from the game and how their country’s resources and goals affected how they played the game.

### 1.3. Study Objective

The overall objective of this study was to (1) describe the structure of a global health elective for pharmacy students, and (2) assess educational outcomes related to knowledge and attitudes associated with global health. Outcomes were evaluated through course assessments as well as a survey questionnaire regarding knowledge (perceived and formal) and attitudes regarding the course activities and topic areas.

## 2. Methods

### 2.1. Course Assessments

The tropical disease eradication plan and culture/ethics case studies were issued as individual written assignments with a single grade for each. The country health system project was split into two major components: interim submission of the presentation draft (30%) and final presentation in class, as scored by faculty (50%) and student peers (20%). The patient education project included four stages: initial country assessment (20%), plan for project (20%), presentation of idea in class (30%) and final submission of the project (30%). The Game of International Drug Discovery was followed by a reflection post-game.

### 2.2. Survey Questionnaire

In the second year offering of the course, an investigator-designed questionnaire was delivered as a pre-/post-survey at the beginning and end of the semester. Survey items were compiled based on a literature review performed by the investigators; with no validated tool available that met the needs of the study, the investigators worked through multiple iterations of discussion to achieve the final instrument delivered to students. The pre-survey questionnaire assessed respondent characteristics and experience relevant to global health, items on knowledge perception (8 items) and attitudes (7 items) regarding global health and formal knowledge assessment questions (16 items) in domains of global burden of disease, infectious disease, culture/ethics, drug discovery and organizational roles. Knowledge perception and attitudes were assessed using five-level Likert-type items (1 = strongly disagree, 2 = disagree, 3 = neutral, 4 = agree, 5 = strongly agree). A series of open-ended questions was also included to assess student goals for the course. For the post-survey, items on knowledge perception, attitudes and formal knowledge assessment were repeated. Additional questions on course and game outcomes (4 items each) were included for student reflection. The survey was optional to the course with no bearing on formal grade assessments. The survey questionnaire was approved by the Duquesne University institutional review board.

The survey was delivered via Qualtrics (Provo, UT, USA) and analysis was performed using SPSS Statistics 24 (IBM Corp.; Armonk, NY, USA). Descriptive analyses were conducted for each item, and internal consistency reliability for knowledge perception and attitude domains were conducted using Cronbach’s alpha. Formal knowledge questions were scored as percentage correct. Paired *t*-tests were utilized to compare changes in pre-/post-data for knowledge perception, attitudes and formal knowledge. Statistical tests were clarified with Cohen’s d effect sizes where appropriate. Qualitative data from open-ended opinion items were assessed using a content analysis strategy. Two investigators independently identified open coding categories for themes associated with each item after immersion in the data; frequency patterns for said coding were summarized quantitatively.

## 3. Results and Discussion

### 3.1. Results—Course Assessments

Across major course assessments (Table 1), overall performance was strong for the 11 students participating in the elective. For project-based assignments which included formative assessments temporally issued across the project, performance improved over time, likely based on peer and faculty feedback provided at each stage of the assessment.

For reflections regarding the Game of International Drug Discovery, common themes (found in more than 70% of reflections) regarding learning outcomes noted by students included; need to consider a country’s motivations to accomplish goals (*n* = 8, 100%), differences between developing and developed countries (*n* = 7, 87.5%), and the ability to relate to the material better (*n* = 8, 100%). Students realized quickly that despite everyone playing the game together, there were clear differences in what goals they were seeking to accomplish. Topics mentioned in greater than half of the reflections were a better understanding of the collaborative nature of research (*n* = 8, 62.5%) and that working together made achieving goals easier (*n* = 8, 62.5%). Students also mentioned the responsibility of researchers to maintain ethics and help train developing countries (*n* = 8, 62.5%), which was something briefly mentioned in the didactic portion when discussing National Cooperative Drug Discovery Grants (NCDDGs) and International Conservation and Biodiversity Grants (ICBG), demonstrating that they were able to connect the didactic to the active learning portions of this material. Some students found the activity challenging since the topic is not something they have been exposed to regularly; however, they noted gaining first-hand knowledge and skills regarding the negotiations and various motivations encountered throughout the game.

### 3.2. Results—Survey Questionnaire

Out of the total of 11 students enrolled in the elective, 10 provided complete data on the pre-survey and post-survey. The cohort included seven males (63.6%), with all students having had previous community pharmacy experience, and six (54.5%) with additional experience in the hospital setting. A total of two students (18.2%) reported either previously living or studying abroad, while five (45.5%) had previously traveled abroad for vacation purposes.

Perception of knowledge regarding areas of global health was relatively low among student at the beginning of the course (Table 2), with perceived knowledge of global health systems and neglected tropical diseases rated lowest. Effect sizes for changes for all knowledge perception items were high (Cohen’s d > 0.8). However, perception of knowledge for each item significantly improved at the end of the course. Among attitudes, students held very positive attitudes regarding global health both at the beginning and end of the course, rendering non-significant changes on these items.

For the formal knowledge assessment (16 items), the mean score on the pre-test was 54.2% (range: 23–69%). Highest rated items were formal knowledge of tropic disease eradication (100%), health as a human right (90.9%) and opinion status of the US healthcare system abroad (90.9%). However, scores on the post-test were similar, at 55.6% (range: 31–63%) (*p* = 0.575 for the comparison). Scores on highest rated items generally persisted for tropic disease eradication (80%), health as a human right (100%) and opinion status of the US healthcare system abroad (90%).

In contrast, post-survey quantitative opinion items were uniformly and similarly rated high (Table 3) for both course outcomes as well as game outcomes. Analysis of the qualitative opinion data regarding what students hoped to gain from the course (in the pre-survey) revealed a desire for increased knowledge/understanding about health globally (*n* = 5, 45.5%) as well as interest in how their role as a pharmacist would relate to global health (*n* = 4, 36.4%). On the post-survey, when asked about what they gained from the course, all students made reference to knowledge gains, most commonly on acquiring perspective (*n* = 6, 54.5%) regarding the diversity of health, healthcare and needs globally.

### 3.3. Discussion

In this initial and preliminary assessment of our global health elective course, significant gains were evident regarding the perception of knowledge students had regarding course topics, complemented by previously established positive attitudes. Success on course instructional activities was demonstrated, but formal knowledge gains as assessed by the pre-post survey did not demonstrate significant improvements.

#### 3.3.1. Global Health Competencies

Global health education across the health disciplines has sustained rapid expansion in recent years, albeit not without growing pains. A literature review of 238 articles in global health education by Liu et al. identified lacking curricular standardization and inclusion of professionals outside of medicine as areas for improvement [6]. Recently, the Consortium of Universities of Global Health (CUGH) has provided some guidance on these issues. This group combines expertise from several professions, including medicine, nursing, pharmacy, dentistry, public health, physical/occupational therapy and psychology. In 2015, CUGH released a list of inter-professional competencies for global health, including eight domains included at the ‘global citizen’ competency level, defined as “competency sets required of all post-secondary students pursuing any field with bearing on global health” [7]. These domains included global burden of disease; globalization of health and health care; social and environmental determinants of health; collaboration, partnering, and communication; ethics; professional practice; health equity and social justice; and socio-cultural and political awareness [7].

Development of our elective course and choice of topics was guided by these defined competency domains. However, other areas were added as they were felt to be specifically relevant for coverage in our curriculum structure. Notably, the competency list is highly clinically focused, and our faculty felt strongly that inclusion of basic science relevant to global health would be a useful addition. Additionally, we chose to include higher domain levels within the CUGH framework, such as capacity strengthening and strategic analysis, which were integrated into our patient education project.

#### 3.3.2. Knowledge and Attitudes

Limited published data is available on other global health course structures and their subsequent assessments within pharmacy curricula for comparison, particularly without inclusion of global field experiences. One relevant example is available from Addo-Atuah et al., who described implementation of a global health elective which utilized team-based learning, projects and online learning [8]. Pre-post surveys from the course demonstrated non-significant improvements in knowledge and attitudes about global health as well as significant improvements in perception of skills development for areas such as grant writing, project planning and management of pharmaceutical services [8]. No major formal assessments of knowledge were conducted, although team-based learning did demonstrate improvement on assessments utilized in the course. Poirer et al. described an interprofessional, online global health course with inclusion of team-based work organized across eight topic-oriented modules [9]. Significant changes were noted across most student-assessed knowledge perceptions before and after the course. No data regarding formal knowledge assessments were reported [9].

Outcomes in the current study broadly mirror those found in these previous analyses, although we additionally attempted to assess formalized knowledge changes. Prior to the course, student perceptions of knowledge and demonstration of formalized knowledge were similarly assessed as low. However, after the course as knowledge perceptions were significantly higher, demonstration of formalized knowledge failed to follow, despite gains in this area being an intended learning outcome for the course. Several aspects may have affected achievement of this outcome. First, students may have over-rated themselves on post-assessments in an effort to justify their course progress and learning [10]. Additionally, the approach used by faculty to deliver content may have focused too heavily on exploration of ideas and concepts, which may focus more heavily on attitudinal gains. Failure to demonstrate formal knowledge may also have been the result of the assessment tool itself, including its ability to comprehensively represent the multifaceted content and scope of the course, but additionally may have resulted from the type of formative assessments utilized throughout the course. Unlike curriculum in the required coursework of our PharmD program, which commonly utilizes quizzes and examinations, we chose to approach assessment in this elective course through essays, projects and presentations. Therefore, expectation of improved formal knowledge demonstrated through the post-survey (which utilized a quiz/examination format) may have not been supported through the assessment design used in the course. Additionally, most global health education, particularly in pharmacy education [11,12], is focused in the experiential realm through short-term elective or medical missions abroad. These types of experiences may allow for enhanced reinforcement and application of global health that we were unable to realize within the confines of our didactic elective.

For attitudes, our study failed to demonstrate any differences between pre- and post-assessments. This was primarily the result of high ratings across all items in the pre-survey, and therefore limited opportunity for improvement. We hypothesize the main influence on this result is the elective nature of the course, which self-selected individuals with interest and enthusiasm for this area of study. If instead the course had been a part of the required didactic curriculum, assessments of attitudes might have demonstrated more meaningful results.

#### 3.3.3. Game Learning

A literature review regarding the use of games in pharmacy education and their overall impact on students by Aburahma and Mohamed discussed the benefits and challenges of using games in the classroom, with most game use in pharmacy curricula involving the use of trivia-type games for review of didactic material [13]. Pharmacy education involves a large use of simulation in the clinical aspects of pharmacy for pharmacist patient interaction and evaluation of student pharmacists “soft skills” and practice readiness [14]. The use of simulations can be considered “serious gaming” but has historically been relegated to the clinical aspects of pharmacy education, yet has been shown to increase student motivation and when used properly increase student retention and understanding [15].

For this course, the corresponding author designed the Game of International Drug Discovery to utilize simulation/role-play to enhance student understanding of the complex relationships and motivations involved in international drug discovery collaborations. This topic is a good fit for game learning, due to its complexity and involvement of relationships and interactions which can be simulated in a condensed format in the classroom. One of the most significant advantages of using games is the increased engagement and excitement generated by being actively involved [13]. The topic of international collaboration would be difficult to generate interest and excitement without role-playing as the students would have no frame of reference or stake in the concepts. By allowing the students to represent countries with different resources and goals, they can rise to the understanding of differential motivations and aspirations on their own, as was shown as a common theme through their post-game reflections. When used in the proper context and developed purposefully, serious games can enhance the learning and comprehension of complex topics for students.

#### 3.3.4. Study Limitations

Several limitations are worth mentioning for the current analysis. First of all, a very small sample size was utilized, which renders the data limited in scope and not generalizable across all curricula. We additionally used an investigator-designed survey questionnaire which was non-validated. This was primarily due to limited available literature and instruments measuring the constructs we desired to assess. Due to this, we measured the internal consistency reliability for our attitude and knowledge perception items and found an adequate coefficient of consistency. Finally, we previously mentioned the potential limitation resulting from utilization of a qualitative formal knowledge assessment (multiple choice items), which was different than the assessment methods utilized during the course (essays and projects).

#### 3.3.5. Plans for Future Course Improvements

Based on these initial assessments of the course and alongside other faculty motivations, several changes are anticipated for future offerings. At current time, the patient education project has initiated collaboration with partners in Haiti through support of educational initiatives. Faculty are actively planning to incorporate a ‘spring break-away’ component to the course that will allow opportunity for students to travel to Cap-Haitien for a week for implementation of their projects and to provide longitudinal support for pharmacy services in the area. A key focus of this development has been long-term investment and building a sustainable partnership. Alongside this, at our university, a movement for creation of a global health minor/concentration is gaining traction, which will significantly increase opportunity for students not only to participate in global health education within the field of pharmacy, but also through inter-professional activities.

## 4. Conclusions

Our initial offering of our global health elective for pharmacy students was designed to expose students to a new area of practice through inclusion of projects, multidisciplinary topics, team-based work and community involvement. Students entered the course with positive attitudes, and knowledge perceptions regarding global health increased significantly as a result of the course. However, increased focus is needed for measuring and sustaining formal knowledge gains. Notably, our use of a novel game method was effective for engagement within the area of international drug discovery.

## Figures and Tables

**Table 1:** Performance on course assessments.

Course Assessment	Type	Score, Mean (SD)
Tropical disease eradication plan	Team	93.4 (1.3)
Culture/ethics case studies	Individual	89.6 (6.6)
Country health system project	Individual	
Interim		89.7 (9.2)
Final		92.2 (4.1)
Patient education project	Team	
Country assessment		86.4 (10.7)
Plan for project		90.5 (5.0)
Presentation		95.5 (14.4)
Final		96.1 (4.5)

**Table 2:** Pre-post comparison of knowledge perception and attitudes regarding global health.

Statement	Pre-Score, Mean (SD) *	Post-Score, Mean (SD) *	Mean Diff	*p*-Value
*Knowledge perception* ^†^				
I feel knowledgeable regarding the general topic and goals of global health	2.8 (0.92)	4.8 (0.42)	2.00	<0.001
I feel knowledgeable about other countries’ healthcare systems	1.3 (0.48)	4.3 (0.68)	3.00	<0.001
I feel knowledgeable about neglected topical diseases	1.9 (1.1)	4.7 (0.48)	2.80	<0.001
I feel knowledgeable regarding current issues in global infectious disease	2.4 (0.70)	4.7 (0.48)	2.30	<0.001
I feel knowledgeable regarding cultural influences on health	2.9 (1.1)	4.7 (0.48)	1.80	0.001
I feel knowledgeable regarding drug discovery on the global scale	2.1 (1.1)	4.4 (0.52)	2.30	<0.001
I feel knowledgeable regarding how organizations provide volunteer clinical care abroad	2.8 (1.1)	4.5 (0.53)	1.70	0.003
I feel knowledgeable about roles/opportunities for pharmacists in global health	3.3 (0.82)	4.6 (0.52)	1.30	0.004
*Attitudes* ^‡^				
I believe that global health should be considered an important issue to all pharmacists	4.7 (0.5)	4.7 (0.5)	0	0.999
I believe that ‘thinking globally’ improves the quality of care I can deliver	4.6 (0.73)	4.7 (0.5)	0.11	0.681
I believe that an experience abroad improves a pharmacist's perspective of patient care	4.7 (0.5)	4.7 (0.5)	0	0.999
I believe that I can be involved in global health initiatives without traveling abroad	3.9 (1.17)	4.4 (1.01)	0.56	0.139
I believe that health issues primarily affecting people outside of the USA are still relevant	4.6 (0.73)	4.8 (0.44)	0.22	0.347
I believe that learning about a patient’s culture, religion and beliefs is important to delivering care	4.8 (0.44)	5.0 (0.0)	0.22	0.169
I believe that there is much for the USA to learn about health from around the globe	4.8 (0.44)	4.8 (0.44)	0	0.999

* 1 = strongly disagree, 2 = disagree, 3 = neutral, 4 = agree, 5 = strongly agree; ^†^ Cronbach’s alpha = 0.537 (pre), 0.799 (post); ^‡^ Cronbach’s alpha = 0.900 (pre), 0.747 (post).

**Table 3:** Post-survey opinions regarding the course and The Game of International Drug Discovery.

Statement	Score, Mean (SD) *
*About the course* ^†^	
This course helped me to discover new areas of health I have not previously considered	4.6 (0.52)
This course helped me to develop a widened world view of health	4.7 (0.48)
This course helped me to understand the role of a pharmacist in global health	4.7 (0.48)
This course helped me to better focus my counseling to the needs of each unique patient	4.8 (0.42)
*About the Game of International Drug Discovery* ^‡^	
The game helped me to understand the drug discovery process better	4.6 (0.70)
The game helped me to understand international collaborations in drug discovery	4.7 (0.68)
The game helped me to understand the importance of biodiversity	4.7 (0.68)
The game helped me to understand the differing motivations of nations with respect to drug discovery	4.7 (0.68)
The game helped me to understand the drug discovery process better	4.6 (0.70)

* 1 = strongly disagree, 2 = disagree, 3 = neutral, 4 = agree, 5 = strongly agree; ^†^ Cronbach’s alpha = 0.937; ^‡^ Cronbach’s alpha = 0.986.

## Appendix A. Course Learning Objectives

### Appendix A.1. Learning Objectives

1. Build upon public health principles to identify and discuss global burden of disease
2. Discuss the implications of immigration and movement around the globe upon healthcare infrastructure and delivery
3. Identify and describe current priorities in health affecting the world and how these are applicable to practice in the USA
4. Describe past and present epidemics and strategies created to protect against the future
5. Compare/contrast infrastructures of various healthcare systems and discuss the pros/cons associated with each
6. Discuss the place of ethics and cultural competency in global health and its importance to daily pharmacy practice
7. Identify regulations and respect for autonomy and ownership of biodiversity of different countries and people
8. Describe drug discovery for neglected diseases with a focus on compounds found and aspects of key chemistry

## Appendix B. Game of International Drug Discovery

### Appendix B.1. Game Rules and Description

In this game, players will be split into six teams representing six countries that have different levels of resources and motivations for how to win the game. As a country, a team’s goal is to complete the tasks assigned to you. Every country could accomplish their goals (everyone wins) or no country could accomplish their goals (everyone loses).

There are three main characteristics that each country needs to worry about: (1) money; (2) biodiversity; and (3) science. Money can be used to buy drugs that have already been discovered, explore biodiversity for new drugs, or to invest in science points. Science points determine the number of actions that you may take on your turn. Biodiversity is a natural resource that countries may choose to protect, while others may want to exploit for gains in money and science.

Throughout the game, each country will know what the other country’s resources are but will not know what other country’s goals to win the game are. Each country will have different goals and objectives they must meet to “win”. For example: a country could be told to earn over a specific amount of money and discover at least two cancer drugs OR a country could be told to earn over a specific amount of money but must maintain a certain level of biodiversity.

On each turn, there are three actions a player may take:

▪ Invest in science—purchase additional science points (up to five) for use on future turns
  ○ Prices: 2nd point—$25; 3rd point—$75; 4th point—$150; 5th point—$300
▪ License a drug—negotiate with another country to trade money or biodiversity points for one drug card
▪ Explore biodiversity—explore your own or another country’s biodiversity
  ○ Exploring your own biodiversity is free, and you will keep all profits, drugs and biodiversity points from any discovery
  ○ Exploring another country’s biodiversity requires you to pay them $10 and agree to how to split royalties from any discoveries, with agreements remaining in place until the end of the turn.

Agreements regarding biodiversity must include how to divide money earned, drugs discovered and biodiversity recovered. The countries may each make one counter-offer in setting up the agreement. If an agreement is not reached, then the science point is used and the turn is over. If an agreement has been made, the country exploring will roll a die.

▪ Rolls of 1, 3 or 5 lead to drawing of a “drug” card
▪ Rolls of 2, 4 or 6 lead to drawing of a “nature” card

Available drug cards include:

▪ No activity found—restore biodiversity (9 cards)
▪ Drug recall—−1 drug discovered (3 cards)
▪ Discover an antibiotic—$50 and +1 biodiversity pt (6 cards)
▪ Discover an anticancer drug—+$100 (8 cards)
▪ Discover a neglected tropical disease (NTD) drug—+$20 and +2 biodiversity pt (6 cards)

Available nature cards include:

▪ National park—+1 biodiversity pt (3 cards)
▪ Natural disaster—−1 biodiversity pt (3 cards)
▪ Scientific breakthrough—+1 science pt (3 cards)
▪ Scientific grant—×2 benefits when working with this country for next two turns (6 cards)
▪ Vaccine—protection from diseases outbreak (single use) (6 cards)
▪ Cancer epidemic—if no anticancer drug, −1 science pt (4 cards)
▪ Bacterial outbreak—if no antibiotic, −25% of money (4 cards)
▪ NTD outbreak—If no NTD drug, −1 biodiversity pt (4 cards)

The game will play for six rounds or until time has expired. A running total of resources will be kept on the board (half points for biodiversity and money are allowed, but not for science). Upon completion of the game, each team will reveal their goals and if they accomplished them.

## Country Profiles

**Country**	**Country Type**	**Money**	**Science Points**	**Biodiversity Points**
1	Developed	$200	3	1
2	Developed	$100	3	3
3	Developing	$100	2	3
4	Developing	$50	2	5
5	Underdeveloped	$20	1	8
6	Underdeveloped	$10	1	9

## Country Goals

1. End the game with >$500 and discover 5 drugs
2. End the game with >$300, 4+ biodiversity points and discover 1 of each type of drug
3. End the game with 4+ biodiversity points, 4+ science points and discover 2 antibiotics
4. End the game with >$400, 3+ science points and 6+ biodiversity points
5. End the game with 4+ science points and discover 1 NTD drug
6. End the game with >$100, 8+ biodiversity points and 3+ science points
